# High fat diet-induced obesity and gestational DMBA exposure alter folliculogenesis and the proteome of the maternal ovary^†^

**DOI:** 10.1093/biolre/ioae070

**Published:** 2024-05-30

**Authors:** Gulnara Novbatova, Isabelle Fox, Kelsey Timme, Aileen F Keating

**Affiliations:** Department of Animal Science, Iowa State University, 806 Stange rd, Ames, IA 50011, United States of America; Department of Animal Science, Iowa State University, 806 Stange rd, Ames, IA 50011, United States of America; Department of Animal Science, Iowa State University, 806 Stange rd, Ames, IA 50011, United States of America; Department of Animal Science, Iowa State University, 806 Stange rd, Ames, IA 50011, United States of America

**Keywords:** ovary, obesity, DMBA, gestation, follicle loss

## Abstract

Obesity and ovotoxicant exposures impair female reproductive health with greater ovotoxicity reported in obese relative to lean females. The mother and developing fetus are vulnerable to both during gestation. 7,12-dimethylbenz[a]anthracene (DMBA) is released during carbon combustion including from cigarettes, coal, fossil fuels, and forest fires. This study investigated the hypothesis that diet-induced obesity would increase sensitivity of the ovaries to DMBA-induced ovotoxicity and determined impacts of both obesity and DMBA exposure during gestation on the maternal ovary. Female C57BL/6 J mice were fed a control or a High Sugar High Fat (45% kcal from fat; 20% kcal from sucrose) diet until ~30% weight gain was attained before mating with unexposed males. From gestation Day 7, mice were exposed intraperitoneally to either vehicle control (corn oil) or DMBA (1 mg/kg diluted in corn oil) for 7 d. Thus, there were four groups: lean control (LC); lean DMBA exposed; obese control; obese DMBA exposed. Gestational obesity and DMBA exposure decreased (*P* < 0.05) ovarian and increased liver weights relative to LC dams, but there was no treatment impact (*P* > 0.05) on spleen weight or progesterone. Also, obesity exacerbated the DMBA reduction (*P* < 0.05) in the number of primordial, secondary follicles, and corpora lutea. In lean mice, DMBA exposure altered abundance of 21 proteins; in obese dams, DMBA exposure affected 134 proteins while obesity alone altered 81 proteins in the maternal ovary. Thus, the maternal ovary is impacted by DMBA exposure and metabolic status influences the outcome.

## Teaser

Alterations to ovarian follicle number and the proteome result from both obesity and DMBA exposure, alone and in combination.

## Graphical Abstract

**Graphical Abstract:**
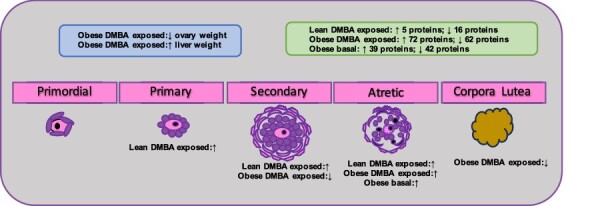

## Introduction

Ovarian function is vital for female reproductive health. Oocytes are the female gametes which develop within a follicular structure surrounded by somatic cells. The number of germ cells within primordial follicles that will grow into the follicular pool is limited [1] and folliculogenesis describes activation of oocyte and somatic cell growth and differentiation orchestrated both locally [2] and centrally [1]. Environmental ovotoxicants can impair ovarian function impeding folliculogenesis and/or ovarian steroidogenesis [3–6] and can also destroy follicles at different stages of growth to result in premature ovarian failure (POF) [5] and loss of proper ovarian function increases risk of multiple diseases including osteoporosis [7], hypercholesterolemia [8], neurological [9], and cardiovascular disorders [10].

During pregnancy, maternal exposure to ovotoxicants and/or endocrine disrupting chemicals could negatively affect fetal development leading to a broad spectrum of health complications or increased susceptibility to metabolic disorders for *in utero* exposed offspring [11–18]. Less is understood regarding the global issue of maternal health, however, pregnant females subjected to environmental chemical exposure have increased risk of hypertension/pre-eclampsia [19] and breast cancer [20]. Maternal morbidity rates are high in the United States suggesting increased anthropogenic chemical pollution [21] and obesity [22–26] as potential culpable factors. Women from socioeconomically disadvantaged backgrounds are considered to be at a higher risk for both of these contributing factors [27, 28].

7,12-dimethylbenz[a]anthracene (DMBA) is a polycyclic aromatic hydrocarbon (PAH) chemical that is released during the burning of organic matter and is a model genotoxicant and ovotoxicant [29]. The link between female cigarette smoking, PAH exposures and accelerated POF is documented [30–35] and ovarian bioactivation of DMBA to a more toxic metabolite has been characterized [36, 37]. In female rats and mice, DMBA destroys ovarian follicles at all stages of folliculogenesis [33, 36–41] and induction of DNA damage [42–45], oxidative stress [46, 47] and apoptosis [47, 48] are documented as modes of PAH-induced toxicity.

The negative impacts of obesity on reproductive health include poor-quality oocytes [49] with increased risk of infertility [50–54], and endocrine dysregulation [55–57]. Mouse models of diet- and hyperphagia-induced obesity both demonstrate abnormal estrous cycles [41, 58], altered folliculogenesis [41, 52, 55, 59] and steroidogenesis [60–62]. Moreover, obesity can affect the ovarian sensitivity to ovotoxicants by altering the abundance of chemical biotransformation and DNA damage response proteins [41, 44, 62–65].

The majority of studies have been performed in adult non-pregnant mice or have examined the impacts of gestational exposure on offspring ovarian outcomes rather than investigating the maternal impacts. To determine an impact of DMBA exposure on the maternal ovary during gestation and an influence of obesity thereon, this study combined DMBA exposure with the physical stressor of gestational obesity to explore the effects of obesity and DMBA exposure on the maternal ovary. Female lean and obese mice were exposed to DMBA and ovaries collected at post-natal day 2. The impacts on ovarian, liver and spleen weight, ovarian follicle number, circulating progesterone (P_4_) and the ovarian proteome were determined.

## Materials and methods

### Animal handling and tissue collection

All animal procedures were approved by the Institutional Animal Care and Use Committee at Iowa State University. Female C57BL/6 J mice aged 5 weeks were obtained from Jackson Laboratories and fed a control (CT; Prolab RMH 1000, LabDiet, St. Louis, MO; *n* = 20) or a High Sugar High Fat (HSHF) diet representative of a human western diet (#D12451; 45% kcal from fat, 20% kcal from sucrose; Research Diets, NJ; *n* = 20). Females are referred to as lean or obese hereon. Four mice on the HSHF diet did not obtain 30% gain and were excluded from the continuation of the study. After ~30% weight gain in the HFHS relative to the CT mice, females from both groups were mated to unexposed C57BL/6 J male mice for one week or until appearance of a vaginal plug. The day of an observed vaginal plug was designated as gestation day (GD) 0.5 and the male was removed from the cage. Body weights were recorded weekly before pregnancy and every 2nd day during the gestation to adjust the DMBA dose. Both lean and obese mice were intraperitoneally injected with vehicle control (corn oil) or DMBA (1 mg/kg diluted in corn oil) for 7 d starting from GD7. This dose of DMBA has been demonstrated previously to be ovotoxic [41]. This time of gestation (GD) coincides with developing germ cells reaching the fetal ovary [66]. Thus, there were four treatment groups: lean control (LC); lean DMBA (LD); obese control (OC); obese DMBA (OD); *n* = 10 per group; two technical replicates. Dams were euthanized at PND2 by CO_2_ asphyxiation followed by cervical dislocation. Blood for serum collection was obtained via cardiac puncture. Liver and spleen were immediately weighed and snap-frozen in liquid nitrogen. Ovaries were either flash frozen in liquid nitrogen or fixed in 4% paraformaldehyde overnight followed by preservation in 70% ethanol for histological analysis.

### Histological analysis

Four ovaries per treatment were randomly selected for hematoxylin and eosin staining. Followed by fixation in 4% paraformaldehyde for 24 h, ovaries were first preserved in 70% ethanol, and then paraffin embedded to be sectioned in 5-micron thickness using a microtome. Every 6th section was mounted on the microscopy slides, and every 12th ovarian section was counted to establish follicular composition. Healthy follicles were counted as previously described [67]. Follicles undergoing apoptosis or necrosis containing the pyknotic bodies and intense eosinophilic staining were designated and counted as atretic.

### Serum P_4_ hormone level quantification

The obtained serum from PND2 dams from the second replicate trial was used to quantify P_4_ at the Ligand Assay & Analysis Core of the Center for Research in Reproduction, University of Virginia with two technical replicates per sample (reportable range = 0.15–40.0 ng/mL).

### LC–MS/MS analysis

Dams’ ovarian tissue homogenates were analyzed by Protein Facility of the Iowa State University Office of Biotechnology using Q Exactive Tandem Mass Spectrometry. After homogenate samples were reduced with dithiothreitol, proteins were treated with iodoacetamide to modify the cysteine groups followed by overnight digestion with trypsin/Lys-C. Afterwards, samples were treated with formic acid and dried in a SpeedVac chamber. C18 MicroSpin Columns (Nest Group SEM SS18V) were used to desalt the samples. The internal control spiked into the peptide samples used was Peptide Retention Time Calibration standard (Pierce part #88320). The peptides were separated using EASY nLC-1200 system with pulled glass emitter 75 μm X 20 cm column (Agilent capillary, part #160–2644-5) packed with a NextAdvance Presssure Injection Cell and UChrom 3-micron material from nanoLCMS Solutions (part #80002) coupled to a Nanospray FlexIon source (Thermofisher Scientific). Q Exactive Hybrid Quadrupole-Orbitrap Mass Spectrometer with an HCD fragmentation cell (Thermofisher Scientific) was utilized for peptide separation and MS/MS analysis. Proteins were identified by MS/MS database as previously described [13, 68].

Protein abundance and fold changes (FC) between treatment groups were analyzed by the Genome Informatics Facility at Iowa State University. Pathways in which altered proteins have functional roles were identified using DAVID v 6.8 software. The false discovery rate (FDR) for pathways was calculated using DAVID v 6.8 software.

### Statistical analysis

Unpaired t-test using Prism 9.0.1 software (GraphPad Prism) were used for data sets that met normal distribution parameters. For the data sets with skewed distributions, Wilcoxon Mann–Whitney comparison was utilized. Statistical significance was defined as *P* < 0.05 with a tendency for biological meaning if *P* < 0.1. Pathway enrichment analyses were generated using proteins identified by LC–MS/MS results for which *P* < 0.1. The results in bar charts represent mean ± standard error of mean (SEM).

## Results

### Effect of obesity on pregnancy outcome

**Figure 1:**
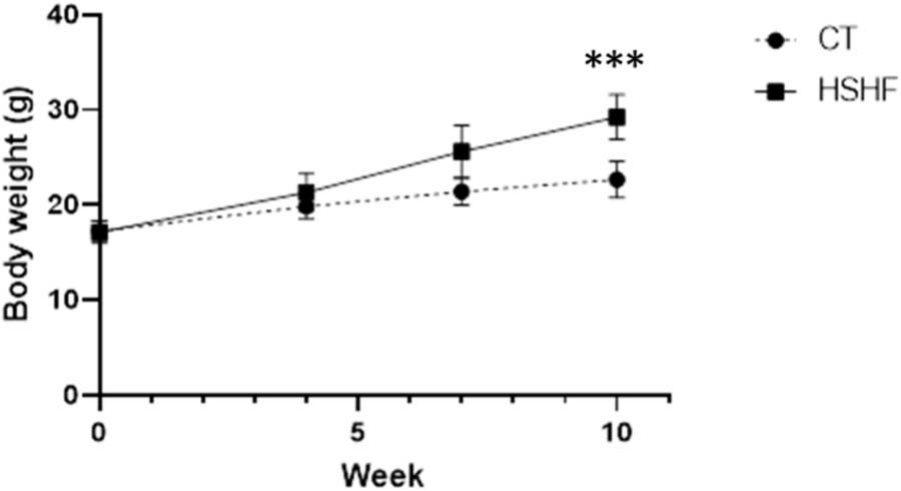
Change in body weight due to CT or HSHF diet prior to conception. Five-week-old female C57Bl/6 J mice were randomly assigned to CT (*n* = 20) or HFHS (*n* = 20; 45% kcal from fat, 20% kcal from sucrose) dietary treatment, and body weights were recorded weekly. Data points represent mean body weight ± SEM for weeks 0, 4, 7, and 10. ^***^P < 0.0001.

Female mice were fed either a CT or HSHF diet for 10 weeks before mating to males who were fed a standard chow diet. Pregnancy success was achieved in 52.5% of females from CT group and 37.5% of females from the HFHS group (data not shown). The mean body weight for females at the beginning and the end of 10 wk of assigned dietary treatment (CT *n* = 19; HSHF *n* = 16) determined that the females on the HFHS diet were heavier than their CT diet counterparts (*P* < 0.0001; Figure 1) prior to mating with males.

### Impact of gestational DMBA exposure on maternal body and organ weight in lean and obese mice

**Figure 2:**
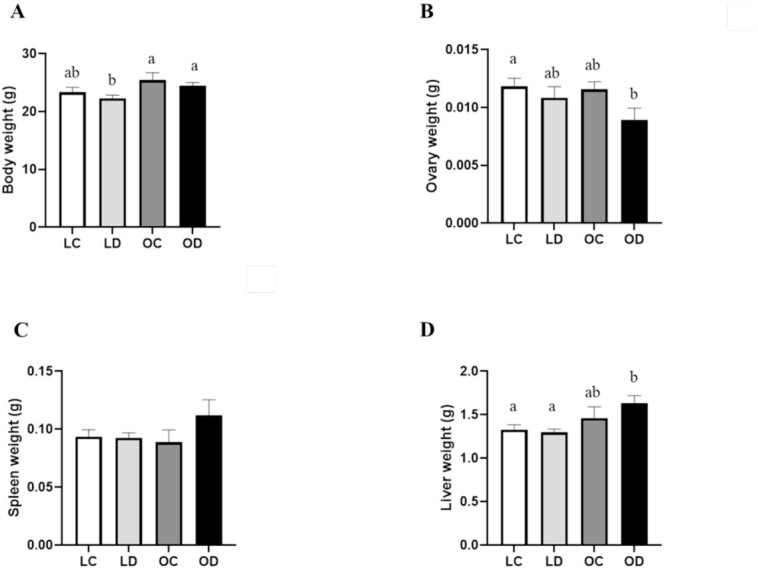
Impact of gestational obesity and DMBA exposure on body and organ weight in postpartum mice. Lean (*n* = 19) and obese (*n* = 16) pregnant dams were intraperitoneally (i.p) dosed with corn oil or DMBA (1 mg/kg) for 7 d starting from GD7. Mice were euthanized at PND2 and (A) body (LC *n* = 9; LD *n* = 12; OC *n* = 6; OD *n* = 9), (B) ovary (LC *n* = 10; LD *n* = 11; OC *n* = 6; OD *n* = 8), (C) spleen (LC *n* = 10; LD *n* = 12; OC *n* = 6; OD *n* = 9), and (D) liver (LC *n* = 9; LD *n* = 12; OC *n* = 6; OD *n* = 9) weights were recorded. Data points represent mean ± SEM. Different letters indicate statistical differences; *P* < 0.05.

Exposure to DMBA did not impact maternal body weight at the cessation of dosing relative to the respective controls; however the lean mice treated with DMBA had lower (*P* < 0.05) body weight compared to both of the obese groups (Figure 2A). There was an additive effect of gestational obesity and DMBA exposure on ovary weight with a decrease (*P* < 0.05) in ovarian weight in OD relative to LC dams, which experienced ~25% loss of ovarian mass (Figure 2B). There was no effect of gestational obesity nor DMBA exposure on maternal spleen weight at PND2 (Figure 2C). Gestational DMBA exposure also did not impact hepatic weight in lean dams, but liver weight was increased in obese DMBA treated dams compared with both lean groups (*P* < 0.05) (Figure 2D).

### Impact of gestational DMBA exposure on circulating serum P_4_ level in postpartum lean and diet-induced obese mice

**Figure 3:**
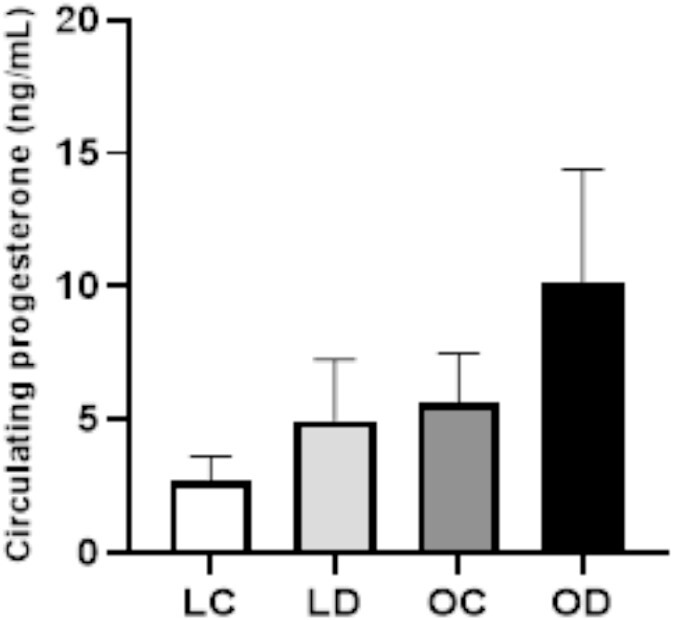
Impact of gestational obesity and DMBA exposure on circulating serum progesterone in postpartum dams. Lean (*n* = 19) and obese (*n* = 16) pregnant dams were intraperitoneally (i.p) dosed with corn oil or DMBA (1 mg/kg) for 7 d starting from GD7. Mice were euthanized at PND2 and progesterone (ng/ml) was measured in serum (*n* = 4 per group). Data points represent mean ± SEM.

The level of P_4_ was measured in the serum obtained from PND2 dams. There was no treatment effect on serum P_4_ (*P* > 0.05; Figure 3) in either lean or obese dams.

### Impact of both obesity and DMBA on the number of follicles in the ovaries from PND2 dams

**Figure 4:**
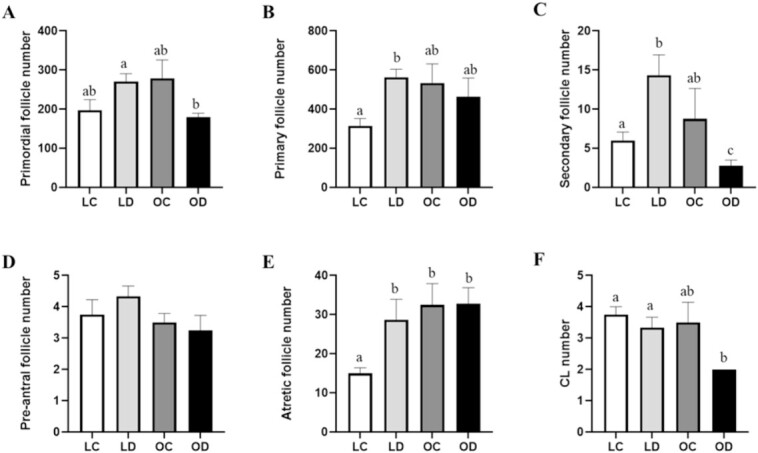
Impact of gestational obesity and DMBA exposure on ovarian follicle composition in postpartum dams. Lean (*n* = 19) and obese (*n* = 16) pregnant dams were intraperitoneally (i.p) dosed with corn oil or DMBA (1 mg/kg) for 7 d starting from GD7. Mice were euthanized at PND2 and (A) primordial, (B) primary, (C), secondary, (D), pre-antral, (E) atretic follicles number, and (F) CL were counted in the ovaries from postpartum dams. Data points represent follicle number mean ± SEM. Different letters indicate statistical differences; *P* < 0.05.

There was no effect (*P* > 0.05) of DMBA exposure on primordial follicle number in lean dams. The number of primordial follicles was decreased (*P* < 0.05) in obese dams exposed to DMBA relative to lean DMBA exposed dams (Figure 4A). In lean mice, DMBA exposure increased the number of primary follicles (*P* < 0.05; Figure 4B) but this was not observed in the obese mice. Exposure to DMBA increased the number of secondary follicles in lean mice, but in obese dams secondary follicle number was decreased (*P* < 0.05; Figure 4C). There was no effect of body composition or DMBA exposure on pre-antral follicle number (Figure 4D). The number of atretic follicles was increased (*P* < 0.05) by both obesity and DMBA exposure (Figure 4E). Ovaries from DMBA exposed obese dams had decreased (*P* < 0.05) numbers of corpora lutea (CLs) compared with ovaries from lean dams (Figure 4F).

### Additive effects of obesity and DMBA exposure on the ovarian proteome in postpartum dams

In postpartum dams, the proteomic analyses of ovarian tissue were compared between four groups: LC vs. LD, LC vs. OC, LD vs. OD, and OC vs. OD (Table 7).

**Table 7:** Number of proteins altered by obesity basally and in the presence and absence of DMBA exposure

Treatment	LC vs. OC	LC vs. LD	OC vs. OD	LD vs. OD
Increased	39	5	72	11
Decreased	42	16	62	10

**Figure 5:**
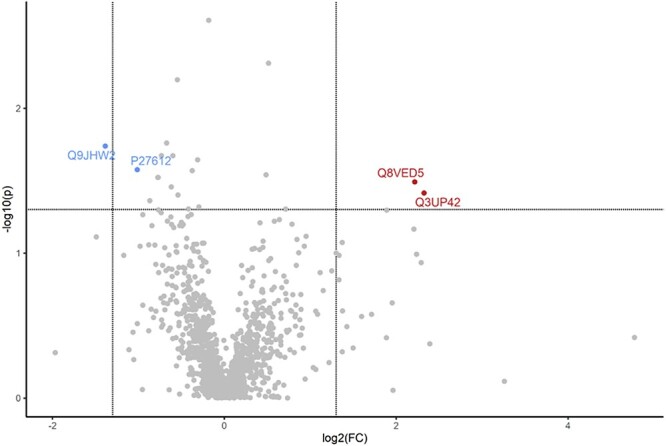
Gestational DMBA exposure alters the ovarian proteome of lean postpartum dams. Lean pregnant dams were intraperitoneally (i.p) dosed with corn oil or DMBA (1 mg/kg) for 7 d starting from GD7. Mice were euthanized at PND2 and protein homogenates analyzed by LC–MS/MS. Bioinformatic analyses were performed to determine differences in protein abundance in lean mice exposed to DMBA relative to vehicle control mice. The dots in the volcano plots above horizontal line indicate proteins that differed in abundance at *P* < 0.05 with dots to the right and left of the vertical lines indicating increased and decreased in abundance, respectively.

Considering the impact of obesity alone (LC vs. OC), HFHS diet-induced obesity changed (*P* < 0.05) the levels of 81 proteins (Table 1). Obesity increased the abundance of 39 proteins and decreased 42 proteins. Out of multiple proteins altered by obesity with FC > 2, RNA-binding motif protein 3 (RBM3) was increased almost 14-fold (Figure 5). Two proteins with roles in detoxification of xenobiotics, Glutathione S-transferase P1 (GSTP) and Quinone oxidoreductase (NADPH:quinone reductase), were decreased by 0.7-fold and 0.4-fold, respectively. There was a total of 13 pathways as possible targets of obesity in postpartum dams recognized by DAVID statistical analysis with FDR ≤ 0.08 including “Glutathione” and “Metabolic” pathways, “Carbon metabolism”, “Thermogenesis”, and “Oxidative phosphorylation” (Table 2).

**Table 1:** Ovarian proteins affected by obesity in postpartum dams (LC vs. OC)

*Protein name*	*Gene name*	*FC*	*log2(FC)*	*P value*
RNA-binding motif protein 3	*Rbm3*	13.67	3.77	0.0026
Barrier-to-autointegration factor	*Banf1*	5.76	2.53	0.0418
Zona pellucida sperm-binding protein 3	*Zp3*	5.64	2.50	0.0457
60S ribosomal protein L10	*Rpl10*	4.11	2.04	0.0233
Protein FAM136A	*Fam136a*	3.99	1.99	0.0392
Non-histone chromosomal protein HMG-17	*Hmgn2*	3.80	1.92	0.0272
RNA-binding protein 8A	*Rbm8a*	3.72	1.90	0.0343
Rpl37a protein	*Rpl37a*	3.68	1.88	0.0345
Parvalbumin	*Pvalb*	3.47	1.80	0.0270
PRKC apoptosis WT1 regulator protein	*Pawr*	3.03	1.60	0.0351
High mobility group protein HMG-I/HMG-Y	*Hmga1*	2.91	1.54	0.0124
Tropomodulin-3	*Tmod3*	2.88	1.53	0.0404
Calpain inhibitor	*Cast*	2.76	1.46	0.0368
Uncharacterized protein	*Hmgn1*	2.59	1.38	0.0366
Apoe protein	*Apoe*	2.47	1.31	0.0024
Activator of 90 kDa heat shock protein	*Ahsa1*	2.47	1.30	0.0112
Clusterin	*Clu*	2.37	1.25	0.0386
ADP/ATP translocase	*Slc25a4*	2.31	1.21	0.0367
Thioredoxin domain-containing protein 5	*Txndc5*	2.12	1.09	0.0275
Lcn7 protein	*Tinagl1*	2.06	1.04	0.0287
ATP synthase-coupling factor 6, mitochondrial	*Atp5pf*	2.06	1.04	0.0418
Cytochrome c oxidase subunit 5B, mitochondrial	*Cox5b*	2.03	1.02	0.0292
Pigpen protein		2.00	1.00	0.0434
Receptor expression-enhancing protein	*Reep5 Dp1*	1.99	0.99	0.0056
Hepatoma-derived growth factor–related protein 2	*Hdgfl2*	1.96	0.97	0.0441
Protein S100-A11	*S100a11*	1.91	0.93	0.0419
Myosin-10	*Myh10*	1.76	0.81	0.0464
Lamin-B1	*Lmnb1*	1.65	0.72	0.0149
Tumor protein D54	*Tpd52l2*	1.65	0.72	0.0351
Apolipoprotein A-I	*Apoa1*	1.63	0.71	0.0497
Sodium/potassium-transporting ATPase subunit alpha-1	*Atp1a1*	1.57	0.65	0.0118
Nucleophosmin	*Npm1*	1.55	0.64	0.0468
Lactoylglutathione lyase	*Glo1*	1.52	0.60	0.0296
TFG protein	*Tfg*	1.50	0.58	0.0454
KH domain-containing, RNA-binding, signal transduction-associated protein 1	*Khdrbs1*	1.46	0.55	0.0386
Splicing factor 1	*Sf1*	1.44	0.52	0.0490
Cbx3 protein	*Cbx3*	1.41	0.50	0.0258
Laminin subunit gamma-1	*Lamc1*	1.32	0.40	0.0261
Histone H2A type 1-F	*Hist1h2af*	1.13	0.17	0.0096
Splicing factor 3b, subunit 3	*Sf3b3*	0.80	−0.32	0.0473
Rab5C	*Rab5c*	0.80	−0.33	0.0343
Glutathione S-transferase P 1	*Gstp1*	0.71	−0.49	0.0288
RuvB-like helicase	*Ruvbl1*	0.71	−0.50	0.0131
Transcriptional activator protein Pur-beta	*Purb*	0.70	−0.52	0.0361
Protein phosphatase 1 regulatory subunit 7	*Ppp1r7*	0.69	−0.53	0.0010
Vinculin (Metavinculin)	*Vcl*	0.69	−0.53	0.0351
Eif2s2 protein	*Eif2s2*	0.68	−0.55	0.0382
tRNA-binding domain-containing protein	*Aimp1*	0.66	−0.61	0.0381
PCI domain-containing protein (Fragment)	*Eif3e*	0.65	−0.63	0.0458
Malate dehydrogenase, cytoplasmic	*Mdh1*	0.64	−0.65	0.0476
Annexin	*Anxa6*	0.63	−0.66	0.0115
Septin	*Septin7*	0.63	−0.66	0.0460
Polyadenylate-binding protein	*Pabp1*	0.63	−0.67	0.0215
Alpha-1,4 glucan phosphorylase (EC 2.4.1.1)	*Pygb*	0.63	−0.67	0.0230
Branched-chain-amino-acid aminotransferase	*Bcat2*	0.62	−0.68	0.0319
Transgelin	*Tagln*	0.61	−0.71	0.0285
Tubulin-folding cofactor B	*Tbcb*	0.61	−0.72	0.0144
Obg-like ATPase 1	*Ola1*	0.61	−0.72	0.0221
Carbonyl reductase	*Cbr3*	0.60	−0.74	0.0374
Casein kinase II subunit alpha	*Csnk2a1*	0.59	−0.76	0.0134
Coatomer subunit delta	*Copd*	0.57	−0.82	0.0026
Presequence protease, mitochondrial	*Pitrm1*	0.56	−0.83	0.0413
Leukotriene A-4 hydrolase	*Lta4h*	0.55	−0.86	0.0291
Thimet oligopeptidase	*Thop1*	0.54	−0.88	0.0149
Chloride intracellular channel protein	*Clic4*	0.54	−0.88	0.0162
Cytosol aminopeptidase	*Lap3*	0.54	−0.90	0.0226
Echinoderm microtubule-associated protein-like 2	*Eml2*	0.52	−0.94	0.0315
Propanoyl-CoA:carbon dioxide ligase subunit alpha	*Pcca*	0.52	−0.94	0.0424
Carbonyl reductase	*Cbr1*	0.51	−0.96	0.0418
Four and a half LIM domains protein 1	*Fhl1*	0.51	−0.97	0.0096
ADP-ribosylation factor 4	*Arf4*	0.51	−0.97	0.0282
Insulin-degrading enzyme	*Ide*	0.50	−1.01	0.0024
Heat shock protein 70–2	*N/a*	0.50	−1.01	0.0299
Chromobox protein homolog 1	*Cbx1*	0.49	−1.03	0.0379
NAD(P)H-hydrate epimerase	*Naxe*	0.46	−1.12	0.0404
Quinone oxidoreductase	*Cryz*	0.43	−1.22	0.0380
Deaminated glutathione amidase	*Nit1*	0.42	−1.27	0.0333
Diphosphomevalonate decarboxylase	*Mvd*	0.41	−1.28	0.0190
Bcl-2-like protein 13	*Bcl2l13*	0.31	−1.68	0.0056
IgG3	*Ighg3*	0.30	−1.76	0.0161
Prostaglandin reductase 1	*Ptgr1*	0.25	−1.99	0.0244

**Table 2:** Ovarian KEGG pathway targets changed by diet-induced obesity in postpartum dams (LC vs. OC)

*Pathway*	*Genes_Found*	*Gene_Pathway*	*FDR*
Glutathione metabolism	5	72	4.82E-04
Metabolic pathways	21	1619	4.82E-04
Carbon metabolism	5	121	4.66E-03
Cardiac muscle contraction	4	87	6.92E-03
Amyotrophic lateral sclerosis	8	369	7.33E-03
Parkinson disease	6	264	0.02
Prion disease	6	268	0.02
Oxidative phosphorylation	4	135	0.02
Huntington disease	6	302	0.03
Chemical carcinogenesis - reactive oxygen species	5	222	0.03
Protein processing in endoplasmic reticulum	4	172	0.05
Thermogenesis	4	231	0.08
Viral carcinogenesis	4	229	0.08

**Figure 6:**
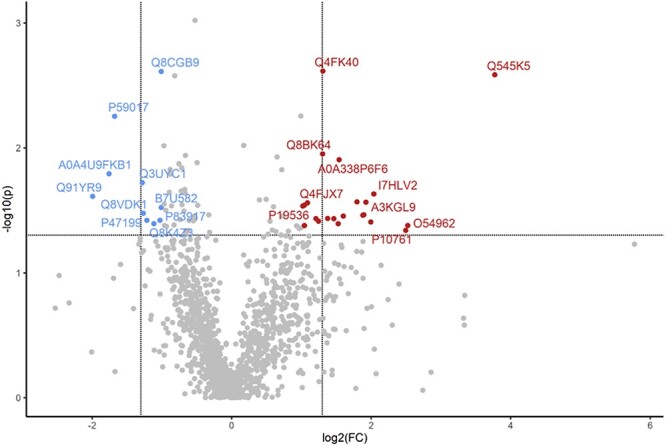
Gestational obesity affects the basal ovarian proteome of postpartum dams. Lean and obese pregnant dams were intraperitoneally (i.p) dosed with corn oil or DMBA (1 mg/kg) for 7 d starting from GD7. Mice were euthanized at PND2 and protein homogenates analyzed by LC–MS/MS. Bioinformatic analyses were performed to determine differences in protein abundance between lean and obese vehicle control treated mice. The dots in the volcano plots above the horizontal line indicate proteins that differed in abundance at *P* < 0.05 with dots to the right and left of the vertical lines indicating increased and decreased in abundance, respectively.

DMBA exposure in lean mice (LC vs. LD) altered (*P* < 0.05) the abundance of 21 ovarian proteins with 5 increased and 16 decreased (Table 3). Of these, only four had a FC >2 in expression: Keratin, Type II cytoskeletal 79 (KRT79) and EF-hand domain-containing protein (S100A9) were increased and (Phospholipase A-2-activating protein [PLA2P] and Omega-amidase [NIT2]) were decreased (Figure 6). In the table for identified pathways, “Gene Found” indicates the number of genes identified from proteomic analyses, and “Gene Pathway” represents the total number of genes in the particular pathway according to Kyoto Encyclopedia of Genes and Genomes (KEGG) pathways database. Pathway analysis of the ovarian altered proteins with *P* < 0.1 identified only “Metabolic pathway” as a possible target with FDR = 0.05.

**Table 3:** Ovarian proteins altered by DMBA exposure in lean postpartum dams (LC vs. LD)

*Protein name*	*Gene name*	*FC*	*log2(FC)*	*P value*
EF-hand domain-containing protein	*S100a9*	5.01	2.32	0.038
Keratin, type II cytoskeletal 79	*Krt79*	4.64	2.22	0.032
PA28 beta subunit	*PSME2b*	1.64	0.71	0.049
Sodium/potassium-transporting ATPase subunit alpha-1	*Atp1a1*	1.43	0.51	0.005
Vitronectin	*Vtn*	1.40	0.49	0.029
Peptidyl-prolyl cis-trans isomerase	*Ppib*	0.88	−0.18	0.002
Electron transfer flavoprotein subunit beta	*Etfb*	0.81	−0.30	0.048
Protein phosphatase 1 regulatory subunit 7	*Ppp1r7*	0.81	−0.31	0.023
Lipoma-preferred partner homolog	*Lpp*	0.77	−0.37	0.027
Peroxisomal trans-2-enoyl-CoA reductase	*Pecr*	0.75	−0.42	0.0495
Cytosol aminopeptidase	*Lap3*	0.69	−0.54	0.040
GTP:AMP phosphotransferase AK3, mitochondrial	*Ak3*	0.68	−0.55	0.006
Peptidyl-prolyl cis-trans isomerase FKBP8	*Fkbp8*	0.66	−0.60	0.021
AP-3 complex subunit beta-1	*Ap3b1*	0.65	−0.62	0.035
DEAH box protein 9	*Dhx9*	0.63	−0.67	0.017
Protein FAM162A	*Fam162a*	0.60	−0.73	0.021
Enoyl-CoA hydratase domain-containing protein 3, mitochondr.	*Echdc3*	0.59	−0.77	0.030
NADH dehydrogenase [ubiquinone] 1 alpha subcomplex subunit 12	*Ndufa12*	0.58	−0.77	0.030
MKIAA0270 protein	*Palm*	0.55	−0.87	0.043
Phospholipase A-2-activating protein	*Plaa*	0.50	−1.01	0.027
Omega-amidase NIT2	*Nit2*	0.38	−1.39	0.018

**Figure 7:**
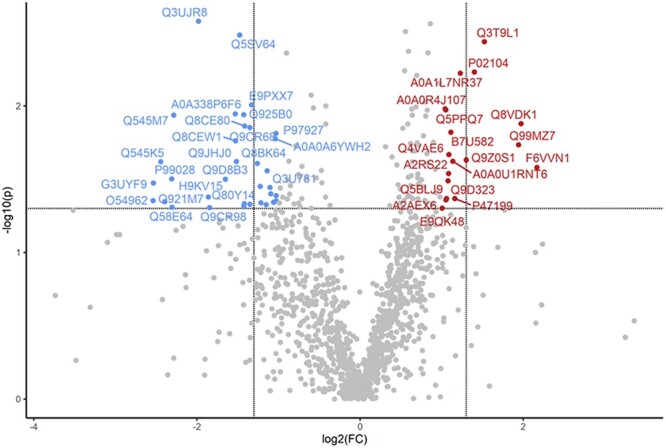
Impact of gestational DMBA maternal exposure on the ovarian proteome of obese dams. Obese pregnant dams were intraperitoneally (i.p) dosed with corn oil or DMBA (1 mg/kg) for 7 d starting from GD7. Mice were euthanized at PND2 and protein homogenates analyzed by LC–MS/MS. Bioinformatic analyses were performed to determine differences in protein abundance between obese mice exposed to vehicle control or DMBA. The dots in the volcano plots above horizontal line indicate proteins that differed in abundance at *P* < 0.05 with dots to the right and left of the vertical lines indicating increased and decreased in abundance, respectively.

DMBA-induced alterations in the ovaries of obese postpartum dams (OC vs. OD) identified the greatest number of altered proteins (total *n* = 134; *P* < 0.05; Table 4). The abundance of 72 proteins were increased, and 62 proteins were decreased. Within these 18 proteins were increased and 34 were decreased with FC > 2 (Figure 7). The same proteins altered in obese control relative to lean control mice that participate in xenobiotic defense were identified with altered levels in the ovaries from DMBA-exposed obese mice: GSTP1 (1.4-fold increase) and CRYZ (2.2-fold increase). In addition, Catalase, an important antioxidant protein had an ~2-fold increase in abundance due to DMBA exposure in the ovaries of obese mice. Caspase 3, an apoptotic cascade protein, was decreased 0.4-fold by DMBA exposure in obese dams. Thirty pathway targets with FDR ≤ 0.08, were identified and 29 of them had an FDR ≤ 0.05 including “Metabolic pathway”, “Carbon metabolism”, “Tight junction”, and “Oxidative phosphorylation” (Table 5).

**Table 4:** Ovarian proteins altered by DMBA exposure in postpartum obese dams (OC vs. OD)

*Protein name*	*Gene name*	*FC*	*log2(FC)*	*P value*
Formin-binding protein 1	*Fnbp1*	4.493	2.168	0.026
Deaminated glutathione amidase	*Nit1*	3.926	1.973	0.013
Peroxisomal trans-2-enoyl-CoA reductase	*Pecr*	3.845	1.943	0.018
Coronin	*Coro1a*	2.875	1.523	0.004
Hemoglobin subunit epsilon-Y2	*Hbb-y*	2.641	1.401	0.006
3′(2′),5′-bisphosphate nucleotidase 1	*Bpnt1*	2.464	1.301	0.023
Arg/Abl-binding protein 2	*Argbp2*	2.343	1.228	0.006
Quinone oxidoreductase	*Cryz*	2.236	1.161	0.043
S-adenosylmethionine synthase	*Mat2a*	2.199	1.137	0.024
Heat shock protein 70–2	*Hsp70–2*	2.163	1.113	0.015
Ras family member A	*Rhoa*	2.126	1.088	0.021
Coronin	*Coro1b*	2.121	1.085	0.029
Signal transducer and activator of transcription	*Stat1*	2.111	1.078	0.032
60S ribosomal protein L27	*Rpl27*	2.085	1.060	0.043
Four and a half LIM domains protein 1	*Fhl1*	2.074	1.052	0.044
Coronin	*Coro1c*	2.071	1.050	0.011
Acyl-peptide hydrolase	*Apeh*	2.060	1.042	0.010
Echinoderm microtubule-associated protein-like 2	*Eml2*	2.01	1.01	0.049
Catalase	*Cat*	1.971	0.979	0.042
Eukaryotic translation initiation factor 3 subunit I	*Eif3i*	1.964	0.973	0.007
NAD(P)H-hydrate epimerase	*Naxe*	1.904	0.929	0.039
ADP-ribosylation factor 4	*Arf4*	1.896	0.923	0.027
Eukaryotic translation initiation factor 5	*Eif5*	1.864	0.898	0.018
60S ribosomal protein L28	*Rpl28*	1.843	0.882	0.047
Proteolipid protein 2	*Plp2*	1.817	0.862	0.003
Glucose-6-phosphate 1-dehydrogenase	*G6pdx*	1.780	0.832	0.004
Isovaleryl-CoA dehydrogenase, mitochondrial	*Ivd*	1.779	0.831	0.025
Uncharacterized protein	*Mta2*	1.769	0.823	0.011
Uncharacterized protein	*Trap1*	1.764	0.819	0.021
Beta-globin	*Hbbt1*	1.747	0.805	0.012
NAD(P)-bd_dom domain-containing protein	*Blvrb*	1.739	0.798	0.045
Acetyl-CoA acetyltransferase	*Acat2*	1.734	0.794	0.044
Obg-like ATPase 1 (GTP-binding protein 9)	*Ola1*	1.733	0.793	0.009
Vacuolar protein sorting-associated protein 35	*Vps35*	1.718	0.781	0.036
60S ribosomal protein L35a	*Rpl35a*	1.713	0.776	0.016
Short-chain specific acyl-CoA dehydrogenase, mitochondrial	*Acads*	1.687	0.755	0.032
Heat shock 70 kDa protein 1A	*Hspa1a*	1.648	0.721	0.034
GTP-binding nuclear protein Ran	*Ran*	1.637	0.711	0.034
Carbonyl reductase [NADPH] 3	*Cbr3*	1.637	0.711	0.043
Tubulin-folding cofactor B	*Tbcb*	1.632	0.707	0.018
Serrate RNA effector molecule homolog	*Srrt*	1.633	0.707	0.029
Uncharacterized protein	*Dhrs4*	1.631	0.706	0.031
Cysteine and histidine-rich domain-containing protein 1	*Chordc1*	1.626	0.701	0.018
Succinate—CoA ligase	*Suclg1*	1.618	0.694	0.006
Coatomer subunit delta	*Copd*	1.606	0.684	0.010
Switch-associated protein 70	*Swap70*	1.587	0.666	0.020
Filamin-C	*Flnc*	1.565	0.646	0.043
Casein kinase II subunit alpha	*Csnk2a1*	1.559	0.641	0.024
Transgelin	*Tagln*	1.558	0.640	0.012
SPI6 (Serine [Or cysteine] peptidase inhibitor, clade B, member 9)	*Serpinb9*	1.553	0.635	0.020
Rab5C	*Rab5c*	1.551	0.633	0.011
Proteasome (Prosome, macropain) 26S subunit, non-ATPase, 11	*Psmd11*	1.543	0.625	0.030
Branched-chain-amino-acid aminotransferase	*Bcat2*	1.536	0.619	0.035
tRNA-binding domain-containing protein	*Aimp1*	1.534	0.617	0.018
Ribosomal protein L15	*Rpl15*	1.523	0.607	0.041
Glyoxalase domain-containing protein 4	*Glod4*	1.508	0.593	0.004
Ribosomal protein L19	*Rpl19*	1.506	0.591	0.031
Protein ORF2	*N/a*	1.499	0.584	0.013
High mobility group protein 1	*Hmgb1*	1.497	0.582	0.042
Thimet oligopeptidase	*Thop1*	1.470	0.556	0.003
PDZ and LIM domain protein 5	*Pdlim5*	1.460	0.546	0.006
MKIAA3012 protein	*Rab1a*	1.454	0.540	0.044
ELAV-like protein	*Elavl1*	1.438	0.524	0.014
Medium-chain acyl-CoA ligase ACSF2, mitochondrial	*Acsf2*	1.437	0.523	0.049
Glutathione S-transferase P 1	*Gstp1*	1.408	0.493	0.037
Vinculin	*Vcl*	1.407	0.493	0.046
Insulin-degrading enzyme	*Ide*	1.392	0.477	0.042
60S ribosomal protein L6	*Rpl6*	1.382	0.467	0.035
Delta(3,5)-Delta(2,4)-dienoyl-CoA isomerase, mitochondrial	*Ech1*	1.367	0.451	0.025
RAB11B protein	*Rab11b*	1.311	0.390	0.022
Heterogeneous nuclear ribonucleoproteins A2/B1	*Hnrnpa2b1*	1.288	0.365	0.048
Predicted gene 20,431	*Gm20431*	1.242	0.312	0.019
Periostin isoform M2	*Postn*	0.774	−0.369	0.048
AHNAK nucleoprotein	*Ahnak*	0.747	−0.420	0.010
Ribosomal protein S14	*rps14*	0.747	−0.421	0.013
Lactoylglutathione lyase	*Glo1*	0.746	−0.423	0.042
Collagen-binding protein	*Serpinh1*	0.722	−0.469	0.033
CCT-beta (T-complex protein 1 subunit beta)	*Cct2*	0.720	−0.475	0.028
Histone H1.1	*H1–1 H1a*	0.689	−0.537	0.031
Glucosamine-6-phosphate isomerase 1	*Gnpda1*	0.672	−0.573	0.010
Nuclear mitotic apparatus protein 1	*Numa1*	0.66	−0.60	0.049978
Sterol O-acyltransferase 1	*Soat1*	0.660	−0.601	0.008
Protein disulfide-isomerase	*P4hb*	0.634	−0.658	0.031
60S acidic ribosomal protein P2	*Rplp2*	0.610	−0.713	0.026
Nucleolin	*Ncl*	0.610	−0.714	0.043
Protein canopy homolog 2	*Cnpy2*	0.601	−0.734	0.049
Tumor protein D54	*Tpd52l2*	0.598	−0.742	0.031
Lysosomal alpha-glucosidase	*Gaa*	0.593	−0.754	0.046
Uncharacterized protein	*Ptma*	0.592	−0.755	0.029
Nucleophosmin	*Npm1*	0.578	−0.790	0.019
High mobility group protein B3	*Hmgb3*	0.571	−0.809	0.047
Cytochrome c oxidase subunit 2	*COX2*	0.553	−0.853	0.035
AAA domain-containing protein	*Psmc3*	0.550	−0.863	0.040
Ubiquitin carboxyl-terminal hydrolase isozyme L3	*Uchl3*	0.546	−0.874	0.024
Uncharacterized protein	*Dctn2*	0.541	−0.887	0.032
CPN10-like protein	*Hspe1-rs1*	0.539	−0.892	0.043
Ubiquitin carboxyl-terminal hydrolase isozyme L1	*Uchl1*	0.535	−0.902	0.004
Multifunctional protein ADE2	*Paics*	0.518	−0.948	0.042
Cytochrome c oxidase subunit 5A, mitochondrial	*Cox5a*	0.508	−0.978	0.036
Inositol-3-phosphate synthase 1	*Isyna1*	0.503	−0.991	0.046
Biglycan	*Bgn*	0.490	−1.028	0.045
Pigpen protein	*N/a*	0.490	−1.029	0.041
Laminin subunit alpha-4	*Lama4*	0.489	−1.032	0.015
Allograft inflammatory factor 1-like	*Aif1l*	0.487	−1.039	0.017
Nucleolar and coiled-body phosphoprotein 1	*Nolc1*	0.480	−1.060	0.045
Y box protein 1	*Ybx1*	0.468	−1.095	0.040
Calumenin	*Calu*	0.465	−1.105	0.036
RRM domain-containing protein	*Srsf3*	0.454	−1.140	0.028
ATP synthase-coupling factor 6, mitochondrial	*Atp5pf*	0.450	−1.152	0.047
Microtubule-associated protein 4	*Map4*	0.430	−1.216	0.046
Glutaredoxin-related protein 5, mitochondrial	*Glrx5*	0.428	−1.224	0.035
Activator of 90 kDa heat shock protein ATPase homolog 1	*Ahsa1*	0.418	−1.259	0.025
Thioredoxin domain-containing protein 5	*Txndc5*	0.397	−1.331	0.010
Caspase-3	*Casp3*	0.392	−1.351	0.047
Cytochrome b-c1 complex subunit Rieske, mitochondrial	*Uqcrfs1*	0.392	−1.352	0.014
Calpain inhibitor	*Cast*	0.375	−1.416	0.014
Complement component 1 Q subcomponent-binding protein, mitochondrial	*C1qbp*	0.374	−1.420	0.046
Serine/arginine-rich splicing factor 7	*Srsf7*	0.372	−1.425	0.048
PRKC apoptosis WT1 regulator protein	*Pawr*	0.372	−1.426	0.011
Myosin-10	*Myh10*	0.359	−1.477	0.003
Tropomodulin-3	*Tmod3*	0.350	−1.517	0.024
Uncharacterized protein	*Hmgn1*	0.347	−1.527	0.017
High mobility group protein HMG-I/HMG-Y	*Hmga1*	0.346	−1.530	0.011
Charged multivesicular body protein 4b	*Chmp4b*	0.318	−1.652	0.032
Protein FAM136A	*Fam136a*	0.278	−1.846	0.049
Protein SON	*Son*	0.275	−1.861	0.042
Transcription factor BTF3	*Btf3*	0.253	−1.981	0.003
Parvalbumin	*Pvalb*	0.205	−2.285	0.012
Elongation factor 1-alpha	*Eef1a1*	0.202	−2.306	0.049
Cytochrome b-c1 complex subunit 6, mitochondrial	*Uqcrh*	0.202	−2.310	0.031
CYFIP-related Rac1 interactor B	*Cyrib*	0.190	−2.395	0.045
RNA-binding motif protein 3	*Rbm3*	0.184	−2.444	0.024
Prefoldin subunit 6	*Pfdn6*	0.173	−2.534	0.034
Barrier-to-autointegration factor	*Banf1*	0.172	−2.538	0.044

**Table 5:** Ovarian KEGG pathway targets affected by gestational DMBA exposure in postpartum obese dams (OC vs. OD)

*Pathway*	*Gene_Found*	*Gene_Pathway*	*FDR*
Carbon metabolism	9	121	5.43E-06
Metabolic pathways	27	1619	2.81E-05
Citrate cycle (TCA cycle)	4	32	0.0004
Prion disease	9	268	0.0014
Bacterial invasion of epithelial cells	5	76	0.0014
Valine, leucine and isoleucine degradation	4	57	0.0034
Non-alcoholic fatty liver disease	6	156	0.0047
Parkinson disease	8	264	0.0047
Amoebiasis	5	107	0.0047
Tight junction	6	167	0.0065
Amyotrophic lateral sclerosis	9	369	0.0078
Oxidative phosphorylation	5	135	0.0104
Spliceosome	5	134	0.0104
Cardiac muscle contraction	4	87	0.0104
Focal adhesion	6	201	0.0134
Measles	5	146	0.0134
Proteoglycans in cancer	6	205	0.0134
Diabetic cardiomyopathy	6	211	0.0150
Regulation of actin cytoskeleton	6	220	0.0181
Thermogenesis	6	231	0.0217
Protein processing in endoplasmic reticulum	5	172	0.0226
Leukocyte transendothelial migration	4	118	0.0226
Salmonella infection	6	253	0.0264
Purine metabolism	4	134	0.0320
Chemical carcinogenesis - reactive oxygen species	5	222	0.0518
Alzheimer disease	7	383	0.0523
Huntington disease	6	302	0.0523
Pathways of neurodegeneration - multiple diseases	8	471	0.0523
Viral carcinogenesis	5	229	0.0529
Endocytosis	5	272	0.0855

**Figure 8:**
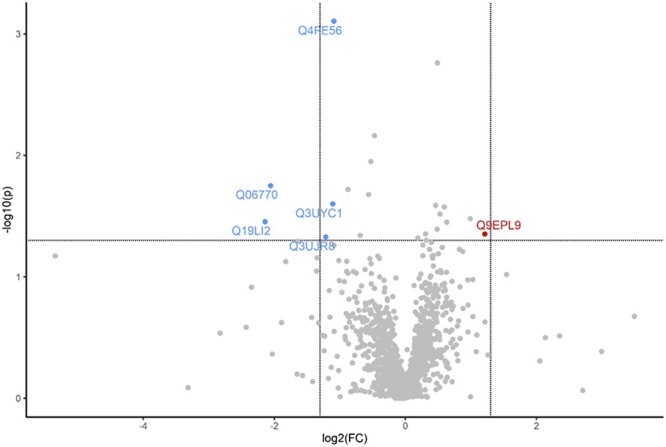
Gestational obesity alters the maternal ovarian proteomic response to DMBA exposure. Lean and obese pregnant dams were intraperitoneally (i.p) dosed with corn oil or DMBA (1 mg/kg) for 7 d starting from GD7. Mice were euthanized at PND2 and protein homogenates analyzed by LC–MS/MS. Bioinformatic analyses were performed to determine differences in protein abundance in the ovaries due to DMBA exposure in lean and obese dams at PND2. The dots in the volcano plots above horizontal line indicate proteins that differed in abundance at *P* < 0.05, with dots to the right and left of the vertical lines indicating increased and decreased in abundance, respectively.

Finally, the differential impacts of DMBA exposure in lean and obese mice were determined (LD vs. OD) and 21 proteins had differential abundance with 11 increased and 10 decreased (*P* < 0.05; Table 6). Peroxisomal-CoA oxidase 3 and coronin proteins were increased ~2-fold, and 5 proteins levels were decreased with FC > 2 (Figure 8). “Metabolic pathway” was identified as an altered pathway (FDR < 0.5) in the ovaries of obese relative to lean DMBA exposed dams.

**Table 6:** Differential ovarian protein abundance due to gestational DMBA exposure in postpartum lean and obese dams (LD vs. OD)

*Protein name*	*Gene name*	*FC*	*log2(FC)*	*P value*
Peroxisomal acyl-coenzyme A oxidase 3	*Acox3*	2.318	1.213	0.044
Coronin	*Coro1b*	1.987	0.990	0.033
Short-chain specific acyl-CoA dehydrogenase, mitochondrial	*Acads*	1.549	0.631	0.036
Metallothionein-1 (MT-1)	*Mt1*	1.509	0.594	0.027
Delta(3,5)-Delta(2,4)-dienoyl-CoA isomerase, mitochondrial	*Ech1*	1.443	0.529	0.030
Proteolipid protein 2	*Plp2*	1.404	0.489	0.002
Phosphoglucomutase-2	*Pgm2*	1.400	0.485	0.041
3-hydroxyisobutyrate dehydrogenase, mitochondrial	*Hibadh*	1.378	0.463	0.026
Malate dehydrogenase, cytoplasmic	*Mdh1*	1.247	0.319	0.049
Long-chain specific acyl-CoA dehydrogenase, mitochondrial	*Acadl*	1.239	0.309	0.044
60 kDa heat shock protein, mitochondrial	*Hsp60*	1.141	0.191	0.048
Cytochrome c1, heme protein, mitochondrial	*Cyc1*	0.721	−0.471	0.007
E2 ubiquitin-conjugating enzyme	*Ube2k*	0.694	−0.527	0.011
Periostin isoform M2	*Postn*	0.678	−0.560	0.021
RRM domain-containing protein	*Srsf3*	0.620	−0.689	0.046
Isopentenyl-diphosphate Delta-isomerase 1	*Idi1*	0.545	−0.876	0.019
Ubiquitinyl hydrolase 1	*Usp9x*	0.470	−1.091	0.001
Diphosphomevalonate decarboxylase	*Mvd*	0.464	−1.108	0.025
Transcription factor BTF3	*Btf3*	0.431	−1.215	0.047
Corticosteroid-binding globulin	*Serpina6*	0.240	−2.057	0.018
Alpha-1B-glycoprotein	*A1bg*	0.227	−2.141	0.035

## Discussion

Ovarian function is critical for both reproductive and general female health. Follicles at different stages of growth can be targeted by ovotoxicants which can results in premature ovarian aging and, subsequently, negatively impact female health [6]. Exposure to environmental chemicals may also contribute to maternal morbidity [69, 70] prompting research to address risks from environmental pollutants on gestational and postpartum health of exposed mothers. The PAH family impair female reproduction [16, 32, 33] and are released by incomplete combustion of organic matter. DMBA is biotransformed to an active, ovotoxic metabolite [36, 37] in the ovary that result in follicle loss [36, 41, 47, 48, 71]. Female offspring exposed *in utero* to DMBA have reduced numbers of oocytes and poor oocyte quality in adult life [72]. However, the maternal ovarian effects of DMBA exposure are not yet fully elucidated.

Obesity is a metabolic disorder that impedes female reproductive function through impaired oocyte quality [49], endocrine dysregulation [55–57] and concomitant infertility [50–54]. In addition, obesity has been shown to associate with maternal mortality [26]. The ovaries of non-pregnant adult obese mice have molecular alterations that suggest enhanced sensitivity to environmental ovotoxicants, including alterations to proteins with roles in DNA repair [43–45, 73], follicular viability [62, 63], steroidogenesis [74], and gap junction communication [64]. This current study queried if obesity during gestation would impact the maternal response to ovotoxicant exposure. Thus, the hypothesis investigated was that diet-induced obesity would impact the ovarian response to gestational DMBA exposure. In addition, the experimental design permitted comparison of the basal obesity impacts on the ovarian proteome during pregnancy.

A negative impact of high-caloric diet on pregnancy outcome was observed with the number of pregnant mice on HSHF diet being reduced by 15% compared with the control diet fed mice. Similar to a previous study in non-pregnant mice [45], there was lack of a DMBA effect on female body weight post-gestation. Though spleen weight did not differ across treatment groups, there was an additive impact of obesity and DMBA exposure on hepatic weight which was increased in DMBA exposed obese mice. Metabolic dysregulation including non-alcoholic fatty liver disease have been reported due to obesity [75–77] and alterations to hepatic xenobiotic biotransformation could further potentiate ovarian dysfunction. In obese DMBA exposed mice, there was also a reduction in ovary weight, potentially demonstrating a higher susceptibility of ovaries from obese dams to DMBA toxicity in pregnant females.

Progesterone is vital for pregnancy maintenance, and its regulation by PGF2α pathway at the end of gestation is important for the initiation of parturition. Therefore, circulating P_4_ was measured in the postpartum dam serum to examine whether DMBA administration and/or metabolic dysregulation during the gestation could adversely affect this hormone’s level but no treatment effect on circulating P_4_ was observed, discounting an endocrine disrupting effect of obesity and DMBA exposure on this hormone parameter.

Follicular composition in the ovaries was affected by both obesity and DMBA exposure. In lean mice, DMBA exposure increased both primary and secondary follicle number, potentially indicating that normal ovarian function, that should be reduced during pregnancy, is being impacted through alterations to follicular growth dynamics. Obesity alone in the absence of DMBA exposure, increased the number of atretic follicles observed—potentially with negative implications for long-term fertility post-partum. In the obese dams that were exposed to DMBA gestationally, decreased secondary follicle number, decreased CL number and increased atretic follicles were noted. All three of these could be negative in terms of pregnancy outcomes, since endocrine disruption could ensue. A disconnect between circulating P_4_ and the reduced number of CL due to DMBA exposure in obese dams could indicate that placental P_4_ production was not affected but this cannot be concluded from this study alone. Overall, follicular composition in the maternal ovary was altered by obesity and DMBA exposure. Measuring local ovarian regulators of folliculogenesis such as anti-müllerian hormone would be a logical consideration for future work.

Ovarian proteomic profiling was examined to further investigate the adverse effects of both DMBA and obesity during the pregnancy. In lean mice, DMBA exposure altered the abundance of 21 proteins and the metabolic pathway was the sole identified target of DMBA. Contrarily, in obese compared to lean mice, 81 proteins were identified as targets of obesity representing 16 pathways including metabolic, glutathione and oxidative phosphorylation pathways. Interestingly, the glutathione (GSH) pathway is documented to be involved in biotransformation of DMBA in the ovary [41, 47, 78–80]. The GST family of enzymes conjugate GSH to toxicants generally facilitating excretion and cell protection [81–86]. In non-pregnant lean mice exposed to DMBA, GSTP was increased in the ovary, while in obese mice, basal levels of GSTP were higher than in lean mice, but the DMBA-induced increase in GSTP noted in lean mice was absent in obese females [41].

There were additive impacts of the combined insults of obesity and DMBA exposure on the ovarian proteome of obese DMBA exposed relative to obese control treated dams resulting in 134 total number of altered proteins and 30 pathways. The levels of two apoptotic proteins, NIT1 and HSP70, and two antioxidative stress enzymes, GSTP and CRYZ (quinone oxidoreductase) were changed and of interest. HSP70 functions along with NF-E2-related factor 2 (NRF2) transcription factor in a protective cellular signaling initiated by oxidative stress [87]. Both GSTP and CRYZ are downstream targets of the NRF2 pathway [88]. Exposure to DMBA increased both GSTP and NRF2 protein in ex vivo cultured ovaries [80, 89], indicating a role for NRF2 signaling in the ovarian DMBA response. Interestingly, there was lower basal GSTP in the ovary of obese mice in the absence of DMBA exposure, but during DMBA exposure in the obese ovary, GSTP was increased. Thus, there are deficits in the ovary of an obese female for GSTP-regulated processes. In terms of HSP70, in the obese ovary, HSP70–2 was reduced basally but increased due to DMBA exposure. This protein is identified in sperm [90, 91] and is proposed to have a role in ovarian cancer [92]. The function of a routine ovarian role for HSP70–2 is currently unclear.

The level of zona pellucida sperm-binding protein 3 (ZP3) protein which is expressed in extracellular matrix surrounding an ovulated ovum was increased by almost 6-fold in obese mice. Highly conserved in many mammalian species, including humans and mice, this protein is essential for the formation of the zona pellucida and successful sperm binding [93]. Human females with missense mutations in the *ZP3* gene are infertile [94, 95] thus altered ZP3 due to obesity might compromise female fertility.

Proteins involved in apoptosis were perhaps unsurprisingly altered by DMBA exposure. Proteins related to cytochrome c function including UGCRH, COX5A, COX2 were decreased in ovaries of obese mice exposed to DMBA. Additionally, pro-apoptotic caspase 3 which was reduced in abundance in obese DMBA-exposed mice, which was unexpected considering the higher levels of atretic follicles observed in both obese and DMBA-exposed mouse ovaries but it is important to consider that the proteomic response was captured at a single timepoint, and protein changes prior to this timepoint could have initiated atresia but may not have been captured herein.

In obese mice exposed to DMBA, three coronin (CORO) proteins were increased >2-fold in the ovary—CORO1A, CORO1B, and CORO1C. Changes in these proteins were not detected in the lean mice exposed to DMBA nor were they evident due to obesity alone. CORO proteins are actin-related proteins [96] and immunodeficiency-related disease in humans have been linked to dysfunction of CORO proteins [97, 98]. Since PAH chemicals are documented as immunotoxicants [99, 100], there is a potential interaction between obesity and DMBA that alters ovarian function related to immune cell function and actin organization. Additionally, CORO1A is implicated in autophagy [101] and cigarette smoke exposure induces autophagy in the ovary as a means of ovotoxicity [35, 102]. The literature on an ovarian-specific CORO protein role is scant, however, use of serum CORO1A as a biomarker of neural tube defects in offspring is proposed [103]. Interestingly, there is increased risk of neural tube defects in offspring from both obese [104, 105] and PAH exposed [106–108] females.

In conclusion, our findings are impactful considering environmental exposure of pregnant women to ovotoxicants and high incidences of obesity in society. The maternal ovary follicle composition findings of both obesity and DMBA exposure are concerning and could result in long-term ovarian function impairments. Moreover, identification of the ovarian proteomic effects of basal obesity and of DMBA exposure in the absence and presence of obesity identified ovarian adverse outcome pathways, and DMBA exposure for one third of gestation caused differential alterations in lean and obese dams. The long-term maternal reproductive consequences cannot be deduced from this study alone but there is potential for impaired subsequent reproduction in ovotoxicant exposed, both lean and obese, dams similar to that previously demonstrated in offspring of obese dams who received a second hit exposure to an obesogenic diet in adulthood [109]. Future work to examine local ovarian folliculogenesis regulators and cellular location in which proteome changes occur are warranted.

## Author contributions

GN performed the animal studies, all analyses on ovarian tissue, assisted in study design, interpreted data and wrote the paper. IF and KT aided in animal handling and tissue collection; AK designed experiments, garnered funding, aided in data interpretation and edited the manuscript.

Conflict of interest: No conflict of interest exists.

## Data availability

Data available upon request.

## References

[1] Hirshfield AN . Development of follicles in the mammalian ovary. Int Rev Cytol 1991; 124:43–101. 2001918 10.1016/s0074-7696(08)61524-7

[2] Liu K , Rajareddy S, Liu L, Jagarlamudi K, Boman K, Selstam G, Reddy P. Control of mammalian oocyte growth and early follicular development by the oocyte PI3 kinase pathway: new roles for an old timer. Dev Biol 2006; 299:1–11. 16970938 10.1016/j.ydbio.2006.07.038

[3] Neff AM , Flaws JA. The effects of plasticizers on the ovary. Curr Opin Endocr Metab Res 2021; 18:35–47. 33997465 10.1016/j.coemr.2021.01.004 PMC8117085

[4] Laws MJ , Neff AM, Brehm E, Warner GR, Flaws JA. Endocrine disrupting chemicals and reproductive disorders in women, men, and animal models. Adv Pharmacol 2021; 92:151–190. 34452686 10.1016/bs.apha.2021.03.008 PMC9743013

[5] Keating AFaH PB . Mechanisms of reproductive toxicity. In: Nassar AF (ed.), Drug Metabolism Handbook: Concepts and Applications, vol. 1. New Jersey: John Wiley and sons; 2009.

[6] Hoyer PB , Keating AF. Xenobiotic effects in the ovary: temporary versus permanent infertility. Expert Opin Drug Metab Toxicol 2014; 10:511–523. 24450963 10.1517/17425255.2014.880690

[7] Kurtoglu-Aksoy N , Akhan SE, Bastu E, Gungor-Ugurlucan F, Telci A, Iyibozkurt AC, Topuz S. Implications of premature ovarian failure on bone turnover markers and bone mineral density. Clin Exp Obstet Gynecol 2014; 41:149–153. 24779240

[8] Lee JS , Hayashi K, Mishra G, Yasui T, Kubota T, Mizunuma H. Independent association between age at natural menopause and hypercholesterolemia, hypertension, and diabetes mellitus: Japan nurses' health study. J Atheroscler Thromb 2013; 20:161–169. 23079582 10.5551/jat.14746

[9] Slopien R . Neurological health and premature ovarian insufficiency - pathogenesis and clinical management. Prz Menopauzalny 2018; 17:120–123. 30356991 10.5114/pm.2018.78555 PMC6196775

[10] Honigberg MC , Zekavat SM, Aragam K, Finneran P, Klarin D, Bhatt DL, Januzzi JL Jr, Scott NS, Natarajan P. Association of Premature Natural and Surgical Menopause with incident cardiovascular disease. JAMA 2019; 322:2411–2421. 31738818 10.1001/jama.2019.19191 PMC7231649

[11] Lumey LH , Ravelli AC, Wiessing LG, Koppe JG, Treffers PE, Stein ZA. The Dutch famine birth cohort study: design, validation of exposure, and selected characteristics of subjects after 43 years follow-up. Paediatr Perinat Epidemiol 1993; 7:354–367. 8290375 10.1111/j.1365-3016.1993.tb00415.x

[12] Choi H , Jedrychowski W, Spengler J, Camann DE, Whyatt RM, Rauh V, Tsai WY, Perera FP. International studies of prenatal exposure to polycyclic aromatic hydrocarbons and fetal growth. Environ Health Perspect 2006; 114:1744–1750. 17107862 10.1289/ehp.8982 PMC1665416

[13] Novbatova G , Timme K, Severin A, Sayadi M, Keating AF. Maternal pre-conceptional glyphosate exposure impacts the offspring hepatic and ovarian proteome. Toxicol Sci 2023; 194:23–37. 37208198 10.1093/toxsci/kfad047 PMC10306405

[14] Novbatova G , Timme K, Severin A, Sayadi M, Keating AF. Pre-conceptional exposure to glyphosate affects the maternal hepatic and ovarian proteome. Toxicol Sci 2022; 190:204–214. 36173347 10.1093/toxsci/kfac098 PMC9702999

[15] Luderer U , Kesner JS, Fuller JM, Krieg EF Jr, Meadows JW, Tramma SL, Yang H, Baker D. Effects of gestational and lactational exposure to heptachlor epoxide on age at puberty and reproductive function in men and women. Environ Res 2013; 121:84–94. 23194642 10.1016/j.envres.2012.11.001

[16] Lim J , Shioda T, Malott KF, Shioda K, Odajima J, Leon Parada KN, Nguyen J, Getze S, Lee M, Nguyen J, Reshel Blakeley S, Trinh V, et al. Prenatal exposure to benzo[a]pyrene depletes ovarian reserve and masculinizes embryonic ovarian germ cell transcriptome transgenerationally. Sci Rep 2023; 13:8671. 37248279 10.1038/s41598-023-35494-w PMC10227008

[17] Weis KE , Thompson LM, Streifer M, Guardado I, Flaws JA, Gore AC, Raetzman LT. Pre- and postnatal developmental exposure to the polychlorinated biphenyl mixture aroclor 1221 alters female rat pituitary gonadotropins and estrogen receptor alpha levels. Reprod Toxicol 2023; 118:108388. 10.1016/j.reprotox.2023.108388. 37127253 PMC10228234

[18] De La Torre KM , Lee Y, Safar A, Laws MJ, Meling DD, Thompson LM, Streifer M, Weis KE, Raetzman LT, Gore AC, Flaws JA. Prenatal and postnatal exposure to polychlorinated biphenyls alter follicle numbers, gene expression, and a proliferation marker in the rat ovary. Reprod Toxicol 2023; 120:108427. 10.1016/j.reprotox.2023.108427. 37400041 PMC10528725

[19] Poropat AE , Laidlaw MAS, Lanphear B, Ball A, Mielke HW. Blood lead and preeclampsia: a meta-analysis and review of implications. Environ Res 2018; 160:12–19. 28938191 10.1016/j.envres.2017.09.014

[20] Rudel RA , Fenton SE, Ackerman JM, Euling SY, Makris SL. Environmental exposures and mammary gland development: state of the science, public health implications, and research recommendations. Environ Health Perspect 2011; 119:1053–1061. 21697028 10.1289/ehp.1002864 PMC3237346

[21] Varshavsky J , Smith A, Wang A, Hom E, Izano M, Huang H, Padula A, Woodruff TJ. Heightened susceptibility: a review of how pregnancy and chemical exposures influence maternal health. Reprod Toxicol 2020; 92:14–56. 31055053 10.1016/j.reprotox.2019.04.004 PMC6824944

[22] Mariona FG . Does maternal obesity impact pregnancy-related deaths? Michigan experience. J Matern Fetal Neonatal Med 2017; 30:1060–1065. 27279358 10.1080/14767058.2016.1199680

[23] Metwally M , Saravelos SH, Ledger WL, Li TC. Body mass index and risk of miscarriage in women with recurrent miscarriage. Fertil Steril 2010; 94:290–295. 19439294 10.1016/j.fertnstert.2009.03.021

[24] Kulie T , Slattengren A, Redmer J, Counts H, Eglash A, Schrager S. Obesity and women's health: an evidence-based review. J Am Board Fam Med 2011; 24:75–85. 21209347 10.3122/jabfm.2011.01.100076

[25] Charnley M , Newson L, Weeks A, Abayomi J. Pregnant women living with obesity: a cross-sectional observational study of dietary quality and pregnancy outcomes. Nutrients 2021; 13:1652. 10.3390/nu13051652 PMC8153277 34068308

[26] Saucedo M , Esteves-Pereira AP, Pencole L, Rigouzzo A, Proust A, Bouvier-Colle MH, CNEMM study group, Deneux-Tharaux C. Understanding maternal mortality in women with obesity and the role of care they receive: a national case-control study. Int J Obes (Lond) 2021; 45:258–265. 33093597 10.1038/s41366-020-00691-4 PMC7752756

[27] Lincoln KD , Abdou CM, Lloyd D. Race and socioeconomic differences in obesity and depression among black and non-Hispanic White Americans. J Health Care Poor Underserved 2014; 25:257–275. 24509025 10.1353/hpu.2014.0038 PMC4830390

[28] Tonne CC , Whyatt RM, Camann DE, Perera FP, Kinney PL. Predictors of personal polycyclic aromatic hydrocarbon exposures among pregnant minority women in new York City. Environ Health Perspect 2004; 112:754–759. 15121521 10.1289/ehp.5955 PMC1241972

[29] Agency for Toxic Substances and Disease Registry (ATSDR) . Toxicological profile for Polycyclic aromatic hydrocarbons. Department of Health and Human Services: U.S; 1995. 38091452

[30] Mattison DR , Nightingale MR. The biochemical and genetic characteristics of murine ovarian aryl hydrocarbon (benzo[a])pyrene) hydroxylase activity and its relationship to primordial oocyte destruction by polycyclic aromatic hydrocarbons. Toxicol Appl Pharmacol 1980; 56:399–408. 7222024 10.1016/0041-008x(80)90074-5

[31] Mattison DR , White NB, Nightingale MR. The effect of benzo(a)pyrene on fertility, primordial oocyte number, and ovarian response to pregnant mare's serum gonadotropin. Pediatr Pharmacol (New York) 1980; 1:143–151. 6287394

[32] Mattison DR , Schulman JD. How xenobiotic chemicals can destroy oocytes. Contemp Obstet Gynecol 1980; 15:157–169.

[33] Mattison DR . Morphology of oocyte and follicle destruction by polycyclic aromatic hydrocarbons in mice. Toxicol Appl Pharmacol 1980; 53:249–259. 6771884 10.1016/0041-008x(80)90424-x

[34] Mattison DR , Thorgeirsson SS. Ovarian aryl hydrocarbon hydroxylase activity and primordial oocyte toxicity of polycyclic aromatic hydrocarbons in mice. Cancer Res 1979; 39:3471–3475. 113091

[35] Gannon AM , Stampfli MR, Foster WG. Cigarette smoke exposure elicits increased autophagy and dysregulation of mitochondrial dynamics in murine granulosa cells. Biol Reprod 2013; 88:63. 23325812 10.1095/biolreprod.112.106617

[36] Igawa Y , Keating AF, Rajapaksa KS, Sipes IG, Hoyer PB. Evaluation of ovotoxicity induced by 7, 12-dimethylbenz[a]anthracene and its 3,4-diol metabolite utilizing a rat in vitro ovarian culture system. Toxicol Appl Pharmacol 2009; 234:361–369. 19027032 10.1016/j.taap.2008.10.009 PMC2666049

[37] Rajapaksa KS , Sipes IG, Hoyer PB. Involvement of microsomal epoxide hydrolase enzyme in ovotoxicity caused by 7,12-dimethylbenz[a]anthracene. Toxicol Sci 2007; 96:327–334. 17204581 10.1093/toxsci/kfl202

[38] Mattison DR . Difference in sensitivity of rat and mouse primordial oocytes to destruction by polycyclic aromatic hydrocarbons. Chem Biol Interact 1979; 28:133–137. 115601 10.1016/0009-2797(79)90120-0

[39] Shiromizu K , Mattison DR. Murine oocyte destruction following intraovarian treatment with 3-methylcholanthrene or 7,12-dimethylbenz(a)anthracene: protection by alpha-naphthoflavone. Teratog Carcinog Mutagen 1985; 5:463–472. 2874631 10.1002/tcm.1770050609

[40] Borman SM , Christian PJ, Sipes IG, Hoyer PB. Ovotoxicity in female Fischer rats and B6 mice induced by low-dose exposure to three polycyclic aromatic hydrocarbons: comparison through calculation of an ovotoxic index. Toxicol Appl Pharmacol 2000; 167:191–198. 10986010 10.1006/taap.2000.9006

[41] Nteeba J , Ganesan S, Keating AF. Impact of obesity on Ovotoxicity induced by 7,12-Dimethylbenz[a]anthracene in mice. Biol Reprod 2014; 90:68. 24501177 10.1095/biolreprod.113.114215 PMC4435232

[42] Daniel FB , Joyce NJ. 7,12-Dimethylbenz[a]anthracene-DNA adducts in Sprague-Dawley and long-Evans female rats: the relationship of DNA adducts to mammary cancer. Carcinogenesis 1984; 5:1021–1026. 6430583 10.1093/carcin/5.8.1021

[43] Rishi JK , Timme K, White HE, Kerns KC, Keating AF. Altered histone abundance as a mode of ovotoxicity during 7,12-dimethylbenz[a]anthracene exposure with additive influence of obesity. Biol Reprod 2023; 110:419–429. 10.1093/biolre/ioad140 PMC10873273 37856498

[44] Ganesan S , Nteeba J, Keating AF. Enhanced susceptibility of ovaries from obese mice to 7,12-dimethylbenz[a]anthracene-induced DNA damage. Toxicol Appl Pharmacol 2014; 281:203–210. 25448685 10.1016/j.taap.2014.10.004 PMC4275104

[45] Rishi JK , Timme K, White HE, Kerns KC, Keating AF. Obesity partially potentiates dimethylbenz[a]anthracene-exposed ovotoxicity by altering the DNA damage repair response in mice. Biol Reprod 2023; 108:694–707. 36702632 10.1093/biolre/ioac218 PMC10106840

[46] Lim J , Lawson GW, Nakamura BN, Ortiz L, Hur JA, Kavanagh TJ, Luderer U. Glutathione-deficient mice have increased sensitivity to transplacental benzo[a]pyrene-induced premature ovarian failure and ovarian tumorigenesis. Cancer Res 2013; 73:908–917. 23135907 10.1158/0008-5472.CAN-12-3636 PMC3548973

[47] Tsai-Turton M , Nakamura BN, Luderer U. Induction of apoptosis by 9,10-dimethyl-1,2-benzanthracene in cultured preovulatory rat follicles is preceded by a rise in reactive oxygen species and is prevented by glutathione. Biol Reprod 2007; 77:442–451. 17554082 10.1095/biolreprod.107.060368

[48] Sobinoff AP , Mahony M, Nixon B, Roman SD, McLaughlin EA. Understanding the villain: DMBA-induced preantral ovotoxicity involves selective follicular destruction and primordial follicle activation through PI3K/Akt and mTOR signaling. Toxicol Sci 2011; 123:563–575. 21785161 10.1093/toxsci/kfr195

[49] Purcell SH , Moley KH. The impact of obesity on egg quality. J Assist Reprod Genet 2011; 28:517–524. 21625966 10.1007/s10815-011-9592-y PMC3158259

[50] Brewer CJ , Balen AH. The adverse effects of obesity on conception and implantation. Reproduction 2010; 140:347–364. 20395425 10.1530/REP-09-0568

[51] Valckx SD , De Pauw I, De Neubourg D, Inion I, Berth M, Fransen E, Bols PE, Leroy JL. BMI-related metabolic composition of the follicular fluid of women undergoing assisted reproductive treatment and the consequences for oocyte and embryo quality. Hum Reprod 2012; 27:3531–3539. 23019302 10.1093/humrep/des350

[52] Skaznik-Wikiel ME , Swindle DC, Allshouse AA, Polotsky AJ, McManaman JL. High-fat diet causes subfertility and compromised ovarian function independent of obesity in mice. Biol Reprod 2016; 94:108. 27030045 10.1095/biolreprod.115.137414 PMC4939738

[53] Metwally M , Li TC, Ledger WL. The impact of obesity on female reproductive function. Obes Rev 2007; 8:515–523. 17868286 10.1111/j.1467-789X.2007.00406.x

[54] Pasquali R , Gambineri A. Metabolic effects of obesity on reproduction. Reprod Biomed Online 2006; 12:542–551. 16790096 10.1016/s1472-6483(10)61179-0

[55] Niño OMS , da Costa CS, Torres KM, Zanol JF, Freitas-Lima LC, Miranda-Alves L, Graceli JB. High-refined carbohydrate diet leads to polycystic ovary syndrome-like features and reduced ovarian reserve in female rats. Toxicol Lett 2020; 332:42–55. 32629074 10.1016/j.toxlet.2020.07.002

[56] Nestler JE . Obesity, insulin, sex steroids and ovulation. Int J Obes Relat Metab Disord 2000; 24:S71–S73. 10.1038/sj.ijo.0801282 10997613

[57] Azziz R , Dumesic DA, Goodarzi MO. Polycystic ovary syndrome: an ancient disorder? Fertil Steril 2011; 95:1544–1548. 20979996 10.1016/j.fertnstert.2010.09.032 PMC3164771

[58] Chakraborty TR , Donthireddy L, Adhikary D, Chakraborty S. Long-term high fat diet has a profound effect on body weight, hormone levels, and Estrous cycle in mice. Med Sci Monit 2016; 22:1601–1608. 27171231 10.12659/MSM.897628 PMC4917314

[59] Wang N , Luo LL, Xu JJ, Xu MY, Zhang XM, Zhou XL, Liu WJ, Fu YC. Obesity accelerates ovarian follicle development and follicle loss in rats. Metabolism 2013; 63:94–103. 24135502 10.1016/j.metabol.2013.09.001

[60] Miller WL , Auchus RJ. The molecular biology, biochemistry, and physiology of human steroidogenesis and its disorders. Endocr Rev 2011; 32:81–151. 21051590 10.1210/er.2010-0013 PMC3365799

[61] Pasquali R , Pelusi C, Genghini S, Cacciari M, Gambineri A. Obesity and reproductive disorders in women. Hum Reprod Update 2003; 9:359–372. 12926529 10.1093/humupd/dmg024

[62] Nteeba J , Ganesan S, Madden JA, Dickson MJ, Keating AF. Progressive obesity alters ovarian insulin, phosphatidylinositol-3 kinase, and chemical metabolism signaling pathways and potentiates ovotoxicity induced by phosphoramide mustard in mice. Biol Reprod 2017; 96:478–490. 28203716 10.1095/biolreprod.116.143818

[63] Nteeba J , Ross JW, Perfield II JW, Keating AF. High fat diet induced obesity alters ovarian phosphatidylinositol-3 kinase signaling gene expression. Reprod Toxicol 2013; 42:68–77. 23954404 10.1016/j.reprotox.2013.07.026 PMC3838664

[64] Ganesan S , Nteeba J, Keating AF. Impact of obesity on 7,12-dimethylbenz[a]anthracene-induced altered ovarian connexin gap junction proteins in female mice. Toxicol Appl Pharmacol 2015; 282:1–8. 25447408 10.1016/j.taap.2014.10.020 PMC4641708

[65] Ganesan S , Nteeba J, Madden JA, Keating AF. Obesity alters phosphoramide mustard-induced ovarian DNA repair in mice. Biol Reprod 2017; 96:491–501. 28203708 10.1095/biolreprod.116.143800 PMC6366544

[66] Pepling ME . From primordial germ cell to primordial follicle: mammalian female germ cell development. Genesis 2006; 44:622–632. 17146778 10.1002/dvg.20258

[67] Flaws JA , Doerr JK, Sipes IG, Hoyer PB. Destruction of preantral follicles in adult rats by 4-vinyl-1-cyclohexene diepoxide. Reprod Toxicol 1994; 8:509–514. 7881202 10.1016/0890-6238(94)90033-7

[68] Cottrell JS . Protein identification using MS/MS data. J Proteomics 2011; 74:1842–1851. 21635977 10.1016/j.jprot.2011.05.014

[69] Lin Y , Zhou L, Xu J, Luo Z, Kan H, Zhang J, Yan C, Zhang J. The impacts of air pollution on maternal stress during pregnancy. Sci Rep 2017; 7:40956. 28098225 10.1038/srep40956 PMC5241869

[70] Boyles AL , Beverly BE, Fenton SE, Jackson CL, Jukic AMZ, Sutherland VL, Baird DD, Collman GW, Dixon D, Ferguson KK, Hall JE, Martin EM, et al. Environmental factors involved in maternal morbidity and mortality. J Womens Health (Larchmt) 2021; 30:245–252. 33211615 10.1089/jwh.2020.8855 PMC7891208

[71] Keating AF , J C, Sen N, Sipes IG, Hoyer PB. Effect of phosphatidylinositol-3 kinase inhibition on ovotoxicity caused by 4-vinylcyclohexene diepoxide and 7, 12-dimethylbenz[a]anthracene in neonatal rat ovaries. Toxicol Appl Pharmacol 2009; 241:127–134. 19695275 10.1016/j.taap.2009.08.012 PMC2783260

[72] Camlin NJ , Sobinoff AP, Sutherland JM, Beckett EL, Jarnicki AG, Vanders RL, Hansbro PM, McLaughlin EA, Holt JE. Maternal smoke exposure impairs the long-term fertility of female offspring in a murine model. Biol Reprod 2016; 94:39. 26764348 10.1095/biolreprod.115.135848

[73] Clark KL , Roach CM, Keating AF. Obesity alters the ovarian DNA damage response and apoptotic proteins. Reproduction 2020; 160:751–760. 33021950 10.1530/REP-20-0070

[74] Nteeba J , Ganesan S, Keating AF. Progressive obesity alters ovarian Folliculogenesis with impacts on pro-inflammatory and steroidogenic Signaling in female mice. Biol Reprod 2014; 91:86. 25143355 10.1095/biolreprod.114.121343 PMC4435031

[75] Vassilatou E . Nonalcoholic fatty liver disease and polycystic ovary syndrome. World J Gastroenterol 2014; 20:8351–8363. 25024594 10.3748/wjg.v20.i26.8351 PMC4093689

[76] Younossi ZM . Non-alcoholic fatty liver disease - a global public health perspective. J Hepatol 2019; 70:531–544. 30414863 10.1016/j.jhep.2018.10.033

[77] Huang TD , Behary J, Zekry A. Non-alcoholic fatty liver disease (NAFLD): a review of epidemiology, risk factors, diagnosis and management. Intern Med J 2019; 50:1038–1047. 10.1111/imj.14709 31760676

[78] Hwang YP , Han EH, Choi JH, Kim HG, Lee KJ, Jeong TC, Lee ES, Jeong HG. Chemopreventive effects of Furan-2-yl-3-pyridin-2-yl-propenone against 7,12-dimethylbenz[a]anthracene-inducible genotoxicity. Toxicol Appl Pharmacol 2008; 228:343–350. 18255114 10.1016/j.taap.2007.12.018

[79] Liu JZ , Zhang BZ, Milner JA. Dietary selenite modifies glutathione metabolism and 7,12-dimethylbenz(a)anthracene conjugation in rats. J Nutr 1994; 124:172–180. 7905918 10.1093/jn/124.2.172

[80] Bhattacharya P , Keating AF. Protective role for ovarian glutathione S-transferase isoform pi during 7,12-dimethylbenz[a]anthracene-induced ovotoxicity. Toxicol Appl Pharmacol 2012; 260:201–208. 22406437 10.1016/j.taap.2012.02.014 PMC3319246

[81] Tew KD . Glutathione-associated enzymes in anticancer drug resistance. Cancer Res 1994; 54:4313–4320. 8044778

[82] Tew KD , Townsend DM. Glutathione-s-transferases as determinants of cell survival and death. Antioxid Redox Signal 2012; 17:1728–1737. 22540427 10.1089/ars.2012.4640 PMC3474190

[83] Townsend DM , Manevich Y, He L, Hutchens S, Pazoles CJ, Tew KD. Novel role for glutathione S-transferase pi. Regulator of protein S-Glutathionylation following oxidative and nitrosative stress. J Biol Chem 2009; 284:436–445. 18990698 10.1074/jbc.M805586200 PMC2610519

[84] Tew KD , Manevich Y, Grek C, Xiong Y, Uys J, Townsend DM. The role of glutathione S-transferase P in signaling pathways and S-glutathionylation in cancer. Free Radic Biol Med 2011; 51:299–313. 21558000 10.1016/j.freeradbiomed.2011.04.013 PMC3125017

[85] Hayes JD , Pulford DJ. The glutathione S-transferase supergene family: regulation of GST and the contribution of the isoenzymes to cancer chemoprotection and drug resistance. Crit Rev Biochem Mol Biol 1995; 30:445–520. 8770536 10.3109/10409239509083491

[86] Hayes JD , Flanagan JU, Jowsey IR. Glutathione transferases. Annu Rev Pharmacol Toxicol 2005; 45:51–88. 15822171 10.1146/annurev.pharmtox.45.120403.095857

[87] Rinaldi Tosi ME , Bocanegra V, Manucha W, Gil Lorenzo A, Valles PG. The Nrf2-Keap1 cellular defense pathway and heat shock protein 70 (Hsp70) response. Role in protection against oxidative stress in early neonatal unilateral ureteral obstruction (UUO). Cell Stress Chaperones 2011; 16:57–68. 20734248 10.1007/s12192-010-0221-y PMC3024087

[88] Baird L , Yamamoto M. The molecular mechanisms regulating the KEAP1-NRF2 pathway. Mol Cell Biol 2020; 40:e00099–20. 10.1128/MCB.00099-20. PMC7296212 32284348

[89] Madden JA , Hoyer PB, Devine PJ, Keating AF. Acute 7,12-dimethylbenz[a]anthracene exposure causes differential concentration-dependent follicle depletion and gene expression in neonatal rat ovaries. Toxicol Appl Pharmacol 2014; 276:179–187. 24576726 10.1016/j.taap.2014.02.011 PMC4077181

[90] Allen JW , Dix DJ, Collins BW, Merrick BA, He C, Selkirk JK, Poorman-Allen P, Dresser ME, Eddy EM. HSP70-2 is part of the synaptonemal complex in mouse and hamster spermatocytes. Chromosoma 1996; 104:414–421. 8601336 10.1007/BF00352265

[91] Rosyada ZNA , Ulum MF, Tumbelaka L, Solihin DD, Purwantara B, Memili E. Implications of sperm heat shock protein 70-2 in bull fertility. Vet World 2022; 15:1456–1466. 35993069 10.14202/vetworld.2022.1456-1466 PMC9375219

[92] Gupta N , Jagadish N, Surolia A, Suri A. Heat shock protein 70-2 (HSP70-2) a novel cancer testis antigen that promotes growth of ovarian cancer. Am J Cancer Res 2017; 7:1252–1269. 28670489 PMC5489776

[93] Wassarman PM , Litscher ES. Female fertility and the mammalian egg's zona pellucida. Histol Histopathol 2024; 18728. 38487866 10.14670/HH-18-728

[94] Männikkö M , Törmälä RM, Tuuri T, Haltia A, Martikainen H, Ala-Kokko L, Tapanainen JS, Lakkakorpi JT. Association between sequence variations in genes encoding human zona pellucida glycoproteins and fertilization failure in IVF. Hum Reprod 2005; 20:1578–1585. 15860499 10.1093/humrep/deh837

[95] Li W , Li Q, Xu X, Wang C, Hu K, Xu J. Novel mutations in TUBB8 and ZP3 cause human oocyte maturation arrest and female infertility. Eur J Obstet Gynecol Reprod Biol 2022; 279:132–139. 36335766 10.1016/j.ejogrb.2022.10.017

[96] Goode BL , Eskin J, Shekhar S. Mechanisms of actin disassembly and turnover. J Cell Biol 2023; 222:e202309021. 10.1083/jcb.202309021. PMC10638096 37948068

[97] Tangye SG , Bucciol G, Casas-Martin J, Pillay B, Ma CS, Moens L, Meyts I. Human inborn errors of the actin cytoskeleton affecting immunity: way beyond WAS and WIP. Immunol Cell Biol 2019; 97:389–402. 30779216 10.1111/imcb.12243

[98] Liu X , Gao Y, Lin X, Li L, Han X, Liu J. The Coronin family and human disease. Curr Protein Pept Sci 2016; 17:603–611. 26916159 10.2174/1389203717666151201192011

[99] Holladay SD , Smith SA, Besteman EG, Deyab AS, Gogal RM, Hrubec T, Robertson JL, Ahmed SA. Benzo[a]pyrene-induced hypocellularity of the pronephros in tilapia (Oreochromis niloticus) is accompanied by alterations in stromal and parenchymal cells and by enhanced immune cell apoptosis. Vet Immunol Immunopathol 1998; 64:69–82. 9656432 10.1016/s0165-2427(98)00116-0

[100] Pallardy M , Mishal Z, Lebrec H, Bohuon C. Immune modification due to chemical interference with transmembrane signalling: application to polycyclic aromatic hydrocarbons. Int J Immunopharmacol 1992; 14:377–382. 1319963 10.1016/0192-0561(92)90167-j

[101] Deng X , Liu B, Jiang Q, Li G, Li J, Xu K. CREBH promotes autophagy to ameliorate NASH by regulating Coro1a. Biochim Biophys Acta Mol Basis Dis 2024; 1870:166914. 37837948 10.1016/j.bbadis.2023.166914

[102] Gannon AM , Stampfli MR, Foster WG. Cigarette smoke exposure leads to follicle loss via an alternative ovarian cell death pathway in a mouse model. Toxicol Sci 2012; 125:274–284. 22003194 10.1093/toxsci/kfr279

[103] Wang Y , Ma L, Jia S, Liu D, Gu H, Wei X, Ma W, Luo W, Bai Y, Wang W, Yuan Z. Serum exosomal coronin 1A and dynamin 2 as neural tube defect biomarkers. J Mol Med (Berl) 2022; 100:1307–1319. 35915349 10.1007/s00109-022-02236-w PMC9402777

[104] Vena F , D'Ambrosio V, Paladini V, Saluzzi E, Di Mascio D, Boccherini C, Spiniello L, Mondo A, Pizzuti A, Giancotti A. Risk of neural tube defects according to maternal body mass index: a systematic review and meta-analysis. J Matern Fetal Neonatal Med 2022; 35:7296–7305. 34219595 10.1080/14767058.2021.1946789

[105] Movva VC , Spangler B, Young AJ, Paglia MJ, Angras K. A retrospective review of the association between maternal body mass index and the risk of congenital anomalies. Congenit Anom (Kyoto) 2024; 64:17–22. 10.1111/cga.12544 37964631

[106] Huang Y , Lin S, Wang C, Pi X, Jin L, Li Z, Wang L, Ren A. Neural tube defects and ZIC4 Hypomethylation in relation to polycyclic aromatic hydrocarbon exposure. Front Cell Dev Biol 2020; 8:582661. 33304900 10.3389/fcell.2020.582661 PMC7701213

[107] Lin S , Ren A, Wang L, Huang Y, Wang Y, Wang C, Greene ND. Oxidative stress and apoptosis in benzo[a]pyrene-induced neural tube defects. Free Radic Biol Med 2018; 116:149–158. 29309894 10.1016/j.freeradbiomed.2018.01.004 PMC5821680

[108] Langlois PH , Hoyt AT, Lupo PJ, Lawson CC, Waters MA, Desrosiers TA, Shaw GM, Romitti PA, Lammer EJ, and the National Birth Defects Prevention Study. National Birth Defects Prevention S. Maternal occupational exposure to polycyclic aromatic hydrocarbons and risk of neural tube defect-affected pregnancies. Birth Defects Res A Clin Mol Teratol 2012; 94:693–700. 22807044 10.1002/bdra.23045 PMC5048886

[109] Clark KL , Talton OO, Ganesan S, Schulz LC, Keating AF. Developmental origins of ovarian disorder: impact of maternal lean gestational diabetes on the offspring ovarian proteome in mice. Biol Reprod 2019; 101:771–781. 31290541 10.1093/biolre/ioz116

